# The Multiple Realizability of Sentience in Living Systems and Beyond

**DOI:** 10.1523/ENEURO.0375-23.2023

**Published:** 2023-11-14

**Authors:** Nicolas Rouleau, Michael Levin

**Affiliations:** 1 Department of Health Sciences, Wilfrid Laurier University, Waterloo, Ontario N2L 3C5, Canada; 2 Department of Biomedical Engineering, Tufts University, Medford, MA 02155; 3 Allen Discovery Center at, Tufts University, Medford, MA 02155; 4 Wyss Institute for Biologically Inspired Engineering, Harvard University, Boston, MA 02215

**Keywords:** cognition, cyborgs, diverse intelligence, hybrots, intelligence, sentience

## Introduction

Brains implement some of the most complex functions of living systems including intelligence, decision-making, learning, and sentience, which is the capacity for subjective experience. To better understand these and other cognitive functions, neuroscientists have meticulously delineated the brain’s microcircuitry and pathways to relate mental phenomena with discrete neural substrates. One of the driving forces behind what has become an ever-expanding literature on functional neuroanatomy is the longstanding assumption that all mental actions and states can be localized, mapped, or otherwise attributed to specific configurations of brain matter. Consistent with this assumption, by selectively damaging or stimulating brain regions, one could suppress or evoke cognitive or behavioral responses that confirmed suspected structure-function relationships. While this approach has not helped explain why mind emerges from matter, its historical success is a crowning achievement for the field of cognitive neuroscience and continues to support clinical research (Gratton et al., 2020; Suárez et al., 2020). However, we now know that an impressive variety of distinct brain morphologies can implement similar mental processes ranging from associative learning to context-dependent decision-making (Lefebvre and Sol, 2008). The same can be said of comparatively simple ganglia (Sarnat and Netsky, 2002), neural explants (Shultz et al., 2017), and tissue engineered neural cultures (Rouleau et al., 2021; Rouleau, 2022; Rouleau et al., 2023). Further support for the generalizability of cognitive function beyond brains, we have learned that several non-neural organisms display response patterns consistent with animal cognition (Boisseau et al., 2016; Smith-Ferguson and Beekman, 2020). And, indeed, we have now engineered artificially intelligent and bio-robotic hybrid systems that display self-organizing cognitive response patterns (DeMarse et al., 2001; Potter et al., 2003). Crucially, fields ranging across technological cognitive augmentation, synthetic bioengineering, and artificial intelligence (AI) are in need of a framework that facilitates research. The field of diverse intelligence seeks deep invariants across agents of widely differing composition and provenance, to dissolve pseudoproblems that arise from superficial binary categories, and remove barriers that prevent the use of powerful techniques across subfields and substrates. Because of developments in conceptual frameworks and technological advances, it has become reasonable to suggest that cognitive processes, of whatever degree of complexity, can be similarly realized by many different kinds of systems, only one of which is called a “brain.” Other candidate systems such as non-neural cells and tissues (Wood, 1992; Armus et al., 2006; Ginsburg and Jablonka, 2009; Murugan et al., 2021), plants (Calvo Garzón and Keijzer, 2011; Segundo‐Ortin and Calvo, 2022), and fungi (Baluška et al., 2021) may exist on a landscape of cognitive potential that extends beyond living organisms to materials, synthetic intelligences, and other unconventional embodiments of mind.

## Cognition Is Multiply Realizable and Substrate Independent

The related concepts of multiple realizability and substrate independence are critical to the deep, and for now unintuitive, task of separating mental functions from brains as their sole generators. A function is multiply realizable if it can be implemented in many different ways (Batterman, 2000), and is substrate-independent if it can be achieved without the contingency of a specific structure, material, or medium (Bostrom, 2003). One example of a function that shares both of these properties is computation. Indeed, machines and biological organisms alike perform computations by radically different mechanisms and physical substrates (Adamatzky, 2019; Roberts and Adamatzky, 2022); however, they also share essential elements that enable the formation of artificial and natural memristors (Hota et al., 2012; Sah et al., 2014; Volkov et al., 2014) or logic gates (Silva-Rocha and de Lorenzo, 2008) and other components of computational systems. While their energy demands, operating temperatures, and time constants may differ, their functional outputs are in many cases indistinguishable. Cognitive functions are currently thought to emerge by means of information processing within nervous tissues. But what if the same mental activities and states can be similarly achieved by information processing within other substrates? The generalizability of cognition is strongly evidenced by observations that non-neural systems can anticipate reinforcements (Rodríguez and Garzón, 2010), avoid conditioned stimuli (Smith-Ferguson et al., 2022), switch between cooperative and competitive strategies as a function of kinship and resource availability (Novoplansky, 2009), and even display mimicry (Roy, 1993; Ngugi and Scherm, 2006; Schaefer and Ruxton, 2009; Frank, 2019).

This functionalist perspective, which rejects the notion that cognitive systems must be made of neural substrates is consistent with current efforts to create neuromorphic computers and artificial intelligences (AIs) that harness the brain’s algorithms and networking principles to implement more efficient computations in silicon and software (Furber, 2016; van De Burgt et al., 2018; Zador et al., 2023). It is also consistent with advances in behavioral science and philosophy of mind, which offer no indication that mind emerges because of any unique magic properties of terrestrial protoplasm. Indeed, while neurons and their aggregates display specialized properties that are necessary for conventional biological systems to exhibit mental functions, neural substrates are not a necessary part of the minimal set of conditions which must be satisfied to implement cognition (Lyon, 2020). When studying the brain, we should not forget that for each newly identified neural mechanism of cognition, there are likely many more ways to implement the same function in diverse systems all around us or yet unknown. Rather than asking what specific substrates underlie a particular cognitive function in a particular organism, it may be practical to ask what kinds of abstracted elements are required to engineer a generic version of the phenomenon.

## Distinctions without Differences Reinforce the Gatekeeping of Cognitive Language

One expected consequence of multiple realizability is the existence of many distinct labels that refer to the same basic function within different systems and at different scales. Consider the ability of molecules, materials, cells, tissues, organisms, or groups of organisms to change their outputs contingent on a history of inputs. The scale-invariant process by which new information can modify the function of a plastic system is assigned the psychological term “learning” when it is observed in animals, while quantitatively indistinguishable processes in single-cell slime moulds or amoebae are still treated as lesser forms of “adaptation” (Dussutour, 2021; Gershman et al., 2021). This is the case despite considerable evidence of both nonassociative and associative learning responses in non-neural organisms and even in molecular networks (Watson et al., 2010; Biswas et al., 2021, 2022). Encouragingly, functions of the immune system and its cells are often described in explicit terms of learning and memory (e.g., memory B cells, immunologic memory, immune conditioning; Graham and Xavier, 2023). However, similar response patterns and processes in materials, such as conditioned hysteresis (Cragg and Temperley, 1955), nanowire-based synaptic networks (Loeffler et al., 2023), colloid-based computing (Roberts et al., 2023), and reverse piezoelectric memory effects (Wu and Wang, 2011), are treated as foreign and distinct. One of the strongest preconceptions that must be overcome is the generic assumption that behavior takes place in the three-dimensional world; however, many new experimental approaches become available when the notion of a problem space is generalized, enabling the study and exploitation of learning and other forms of intelligent navigation in transcriptional space, physiological space, and anatomic morphospace (Fields and Levin, 2022; Levin, 2023; Mathews et al., 2023).

Interestingly, there is comparatively little resistance from scientists when describing the functional outputs of computer software as “learned” even if there is agreement that biological systems realize the same function by different mechanisms and substrates. However, gatekeeping can be expected when discussing decision-making, problem-solving, intelligence, and sentience in contexts that do not involve animal bodies (Balci et al., 2023), even when the terms are operationalized in alignment with consensus definitions of animal cognition and behavior (Kagan et al., 2022, 2023). These distinctions without differences feed a neuro-centric and anthropo-centric paradigm that should be replaced with general frameworks that unify concepts and facilitate new research programs (Bongard and Levin, 2021; Levin, 2022). For example, the practical adoption of the misnomer “neurobiology” as a metaphor to describe the physiological mechanisms underlying complex plant behavior (Calvo, 2016) would not be necessary under a model of cognition that considers its multiple realizability. Indeed, what is special about neuroscience is not its focus on animal neurons (Koshland, 1983), but its general insights into systems with information processing and control at multiple scales that seamlessly link higher-level cognitive states with the molecular events that implement them (Piedimonte and Benedetti, 2016; Sengupta et al., 2016; Mathews et al., 2023). It is important to note that while academic departments, funding bodies, journals, and educational materials make strong distinctions between neuroscience and other fields such as developmental biology, nature makes few such distinctions. The concepts, and laboratory tools of neuroscience are being successfully applied to many somatic cells which are not anatomic neurons (Adams et al., 2014; Friston et al., 2015; Pezzulo and Levin, 2015; Kuchling et al., 2020). If the tools cannot tell the difference, perhaps the distinction is a constraining limitation that should be shed.

Frameworks for cognition that are substrate-agnostic have been proposed (Rosenblueth et al., 1943; Levin, 2019, 2022) and recent discussions (McShea, 2013, 2016; Bongard and Levin, 2021) have started to erode the unsupportable folk categories of “machines” and “real beings” as tools from evolutionary computation, artificial life, synthetic bioengineering, and AI reveal fundamental and substrate-invariant dynamics (Baluška and Reber, 2021; Reber and Baluška, 2021; Baluška et al., 2022, 2023; Watson et al., 2022). The field of diverse intelligence (Lyon, 2006; Baluška and Levin, 2016; Lyon et al., 2021) promises to unify the key points of an organicist perspective (Rosen, 1974; Goodwin, 1978) with a broad view of basic principles of life and mind implemented in inorganic substrates. These ideas are not merely philosophical. Crucially, they are providing testable and empirically valuable advances in areas such as biomedicine (Lagasse and Levin, 2023; Mathews et al., 2023), as they facilitate the porting of tools across artificial distinctions (“disciplines”) that obscure deep invariances between areas of thought and practice.

## The Fundamental Privacy of Mental States Limits Our Access to the Black Box

The implications that arise from the possibility that mental functions can be implemented by many different kinds of cognitive systems beyond brains or even living organisms are profound. If non-neural and nonliving systems express a capacity to learn, anticipate, evaluate risk, control their attention, and make decisions, distinctions between organisms and their environments may blur in the ecological context (Constant et al., 2018). Beyond the view that animals unilaterally act on their environments, we may view these interactions as active exchanges or communications of a sort. Likewise, if subjective experience is a much more common property of nature than is currently assumed, it may be necessary to ask how we might identify the presence of hidden subjects in the world so as to engage with them or fulfill our ethical obligations to their kind. Indeed, if other body tissues support many of the same biochemical and computational processes as the brain (albeit at different time scales and degrees of complexity), our primary lack of awareness of their inner perspective is no more surprising than our lack of direct awareness of the non-verbal hemisphere's inner life or the subjective experiences of others.

Unlike most cognitive functions, sentience represents a uniquely difficult property to detect or recapitulate in any system. Mental states are fundamentally private phenomena that are only ever inferred by the measurement of objective correlates such as behavioral responses (e.g., verbal/written responses, body language, facial expressions, pointing, ambulation, etc.) or by neuroimaging data that can be correlated with subjective reports (i.e., reverse inference; Poldrack, 2006). To date, there are no available methods to directly measure thoughts, experiences, or any other mental acts or states (Overgaard, 2015), the mind is effectively a Black Box. All empirical claims about mental processes, whether in humans or nonhuman animals, rest on the validity of inferential (nondirect) measures, agency is fundamentally a hypothesis made by an observer (which may be a scientist, a conspecific, or a parasitic hacker) of some system (which indeed may be the system itself). As the early behaviorists pointed out, when an animal’s decision-making or attentional capacities are assessed, it is invariably an operationalized behavior that is quantified rather than the mental process itself. Therefore, it is currently the case that sentience cannot be directly measured in practice, and some have even suggested that it may be inaccessible in principle (Overgaard, 2015; Chalmers, 2017). Observable response patterns are our only windows into the Black Box of the mind, which is a significant epistemic and ethical problem because subjective experiences may not accompany all motor activations and the question of whether or not a system is sentient is critical to how we ought to interact with it. Rather than discrete categories spanning mindless reflexes, automatisms, ideomotor responses, and thoughtful actions, it may be more accurate to view these as part of a continuum of mindedness. Existing theoretical frameworks such as dual-aspect monism (Atmanspacher, 2012) and panpsychism (Chalmers, 2015) suggest that subjects and objects are inseparable components or perspectives of the same phenomena, which would accommodate such a continuum.

In our everyday lives, we humans demand a very low burden of proof when assessing sentience in others and the Turing Test (or “Imitation Game”) is a kind of formalization of our willingness to accept behavioral responses (e.g., verbal or written responses) as reliable evidence of mind. Because humans have access to our own subjective experiences, we also use inductive reasoning to attribute mental states to animals based on the degree to which they are similar to us (Urquiza-Haas and Kotrschal, 2015), forming linear hierarchies of species that cannot be confirmed by measurement (Wilkins et al., 2015). The same reasoning is also applied to simulated subjects (Pantelis et al., 2014). Because it is highly unlikely that all instances of sentience in the Universe must be achieved by dint of the same neural circuits and pathways that underlie human sentience on Earth, our reliance on familiar motor patterns and inductive reasoning by similarity will fall short of detecting most sentient systems. However, in the absence of direct measurement, these crude tools may serve as our only means of accessing the Black Box, even if only be inference. If applied consistently and charitably without prejudice, the use of behavioral patterns can continue to serve as a place-holder method to infer cognitive functions including sentience. Double standards that make tenuous distinctions between behavioral responses in humans or nonhuman animals and other potentially cognitive systems are still pervasive. An equal application of standards must be achieved whereby the system’s origin or composition are treated as irrelevant to its cognitive potential, which must be inferred on the bases of observable (quantifiable) response patterns. Because any inference of mental states will rest on some analogy to the behaviors of our only known example (humans), cognitive functions should be strictly operationalized to avoid the temptations of making exceptions from intuition. Specifically, humans are moderately competent at recognizing intelligence in medium-sized objects moving at medium speeds in 3D space; we are not primed to recognize it in unfamiliar problem spaces or at different spatial and temporal scales. Thus, it is critical to develop principled frameworks and experimental approaches to identify unconventional minds beyond what our narrow, evolutionarily-shaped intuitions provide.

## Identifying the Minimal Scale and Complexity of Sentient Systems

As the category of sentient systems becomes more inclusive, it is worth contemplating the minimal scale at which subjective experiences are possible. Despite the recognition of animal sentience at the organismal scale, it remains controversial to suggest that their microscopic parts at the cellular scale display cognitive features. Is sentience exclusively expressed in multicellular systems or is it a fundamental property of cells that only becomes observable within the narrow spatiotemporal scales that converge with human perception? Are single human neurons sentient? If not, at what point do they become part of a sentient aggregate? Because individual cells display response patterns consistent with stimulus discrimination, problem-solving, decision-making, risk assessment, learning, and adaptive rule-encoding (Dener et al., 2016; De la Fuente et al., 2019; Murugan et al., 2021), the possibility of a subjective dimension at the cellular level akin to mental states should be considered. While this approach is not meant to address the Hard Problem of consciousness, the utility of expanding and scaling our expectations according to the system under observation cannot be understated. While we may never know what it is like to be a bat (Nagel, 1980), it may be scientifically valuable to assume that there are many possible systems that it is like to be; specifically, complex agential systems (living and otherwise) for which the most effective prediction and control requires taking seriously the system’s perception, memories, beliefs, and inner perspective about an action landscape, rather than the landscape as we see it (Uexküll, 2010).

As others have suggested (Hameroff et al., 2002; Tuszynski, 2020), it may be worth considering that subcellular systems (microtubules, gene regulatory networks, phospholipid membranes (Scott et al., 2022), and other minimal active matter (Hanczyc et al., 2011) encode the basic rules that give rise to some degree of cognitive functions? Is there an intrinsic importance to electrical signaling (action potentials) or can cells generate the underlying mechanisms of sentience using other signaling modalities (optical, chemical mechanical)? Promising current accounts of sentience as fundamentally related to affect, prioritization of goals, and functional valence (Paulson et al., 2017; Solms, 2019) imply applicability to a wide range of beings, including single cells and the collective intelligence of morphogenetic processes navigating anatomic morphospace (Levin, 2023), all of which share these fundamental aspects of surviving as an agent in the harsh world. Identifying the necessary and sufficient elements of cognitive function will provide explanations that do not require special properties of neural tissues or the invocation of miracles. It is quite possible that sentient systems are abundant in nature but remain unidentified because they operate at scales of time and space that are deeply unintuitive to human observers; recognizing agency in unconventional substrates and operating in unfamiliar problem spaces (Fields and Levin, 2022) is (for reasons of both practicality and ethics) a critical frontier, depending strongly on new tools from the emerging fields of diverse intelligence and AI. Subjective experience may exist regardless of whether a system is fast or slow, small or large, distributed or centralized, and so on (Schwitzgebel, 2015). Regardless, our current reliance on behavioral responses as an inference filter for the attribution of sentience may limit our ability to detect felt states in unexpected places, motivating novel approaches.

## How Bioengineered Systems Can Help Delineate Mechanisms

Over the last century, the field of neuroscience has supported the development of a roadmap toward the delineation of mechanisms underlying cognition. Electrophysiological and optogenetic tools provided unprecedented multiscale control to link the functions of neurons with their aggregates (tissues, organs, and organisms) to better understand how cognitive functions might emerge from molecular events and electrical variations. However, with the emergent recognition of minimal or basal cognitive functions in non-neural systems, investigators across several fields have adopted new tools with which to explore the broader cognitive landscape. Hybrid robots (hybrots) and other closed-loop embodied culture systems are tractable platforms that display features of cognition such as learning and intelligence *in vitro* (Demarse et al., 2001; Bakkum et al., 2007; Kagan et al., 2022). The evidence suggests that when cells and tissues receive sensory feedback and are able to interact with an external world (e.g., motor outputs), they display response patterns that are goal-directed and anticipatory compared with their disembodied counterparts (Baluška and Levin, 2016). In humans, sensory-motor feedback loops can be subjectively experienced, thus representing a type of subject-object coupling. Whether or not other systems embodied with sensory-motor feedback loops display felt states remains unknown because the same functional outcomes can, in principle, be generated without sentience.

Because hybrots involve biological cells instructing robots and simulated bodies, it is now possible to assess behavior *in vitro*, including those classically associated with the inference of animal sentience including avoidance and place preference. Beyond embodied networks, synthetic biology and tissue engineering techniques are enabling the assembly of iterative tissues with customizable function (Rouleau et al., 2023). We can now design and build modular neural circuits to systematically isolate cognition-promoting algorithms and operational principles. Rather than probing the brains of animals, we can now build neural tissues and assess their cognitive potential in a dish. Similarly, the non-neural tissues of developing organisms can be re-arranged into novel architectures, for example to generate Xenobots and other proto-organisms whose morphologic and behavioral competencies cannot be explained by a long history of selection for those traits, enabling us to ask profound questions about novel bodies and novel minds even in the absence of true exobiological examples (Blackiston et al., 2023). With new tools at our disposal, the possibility of understanding diverse and unconventional intelligences steadily increases. What is critical is to abandon prescientific binary notions of natural kinds (sentient vs mechanical, organism vs machine, etc.) that provide only terminological gatekeeping and suppress deep unification of concepts. The future belongs to continuous models of deep invariants that use an engineering framework to ask what kinds of tools can be applied across disciplines to more efficiently (and ethically) predict, control, create, and relate to a truly diverse set of embodiments of mind.

## New Categories of Sentient Systems Demand Inclusive Ethical Frameworks

As we learn more about the multiple realizability of cognitive functions including sentience, it will be necessary to develop new ethical frameworks in consideration of beings who do not share our evolutionary lineage, composition, or provenance. Making distinctions between “mechanical” and “sentient” systems used to be easy. For the vast majority of human history, one could generally rely on a method as simple as tapping on it. If one heard a metallic or wooden thud, and the system did not move or react visibly, one could expect several things: it was going to be rather boring, generate no discernable output, or, after the industrial revolution, perform automatic but repetitive processes. One might conclude that the system was made by a human, offered no tractable internally generated decisions or preferences, and could ethically be disassembled, rebuilt, or destroyed as needed. Even if the system was complex and active, it would be made of passive components. On the other hand, if one felt a soft, better yet, a warm kind of quality, then one could conclude that the system was the product of natural processes, would be composed of living parts (organs and cells) that had all sorts of independent competencies, would likely offer many surprises of physiology and behavior (prediction and control would be best afforded by thinking about what the system had experienced before, its preferences, etc.), and would require some ethical protections (or at least, careful thought about how to relate to it).

Human beings, and societies, have always struggled to maintain ethical behaviors toward others even when it was clear they had minds and shared many important features. The history of in/out-group relationships among modern humans, and our wildly inconsistent treatment of pets, food animals, etc. underscore a willingness to use utterly irrelevant, superficial distinctions to justify classifications into protected or exploited classes of beings. But, at least the rough heuristic of origin story (evolved vs engineered) and composition (cytoplasm vs metal) gave some way to define relationships, based on where a system within phylogeny (most people agree on the relative importance to be given to mammals vs bacteria, for example). But this long-lasting framework is crumbling rapidly. While it may prove to be a painful process, with many disruptive implications across science, technology, and society, it is ultimately an essential one because the firm categories that gave rise to the classic dichotomy were never good natural kinds. A maturing of scientific and social frameworks requires us to find better, more unified perspectives on the world, as science and engineering catch up to ideas explored in science fiction for many decades.

Indeed, some of these issues were raised by early technologies such as automatons during the middle ages, debates about the status of nonhuman animals in Descartes’ work and others, and about the status of plant and animal chimeras and hybrids not present in the original Garden of Eden. One of the reasons Darwinian evolution was (and remains) so shocking in some quarters is that it emphasizes the continuous relationships between life forms which make it very difficult to draw any scientifically-supportable sharp line corresponding to crisp, binary categories. A related continuum is highlighted by developmental biology, which likewise offers no sharp line at which any interesting aspect (mind, sentience, etc.) snaps into being. But these single-axis continua (on evolutionary, and ontogenic timescales) are just the tip of the iceberg. Recent advances in bioengineering make it clear that the space of possible bodies and minds is astronomically vast, going far beyond the singular history of life on this planet (Darwin’s “Endless Forms Most Beautiful”; Clawson and Levin, 2023). Our future is inevitably going to include co-existence with a very wide diversity of forms on the landscape of cognitive potential that include organisms, cyborgs, hybrid robots, artificial or synthetic intelligences, bioengineered beings, and many unconventional intelligences with both hardware and software components (and that will be the case even if we never find exobiological agents to add to this list).

How are we to make sense of, and relate to, beings that are nowhere on the natural web of life with us? How to think about systems and agents with radically different origin stories and composition? There are yet no clear answers, but what is clear is that What you look like, and How you got here, are no longer viable paths to scientifically and morally justifiable strategies. At stake are numerous fields of science, engineering, and everyday life that fall roughly into at least three categories. (1) Basic science, and a search for the most unified (parsimonious) framework with which to understand mind, its relationship to bodies, its evolutionary origins, and the space of possible beings (Sloman, 1984). (2) Biomedicine and engineering, which seek frameworks for identifying the most efficient set of approaches, ranging from direct engineering to tools from behavioral science, to optimally repair, modify, and create systems like complex bodies, synthetic biobots, and traditional robotics (Pezzulo and Levin, 2015; Davies and Levin, 2023). (3) Ethics, which must mature, so as to do away with distinctions not based on scientific natural kinds, and provide ways for individuals and societies to rationally and compassionately relate to beings that may not look familiar or recognizable by widespread ethical frameworks first developed in prescientific ages.

## Synthesis

Reviewing Editor: Christophe Bernard, INSERM & Institut de Neurosciences des Systèmes Decisions are customarily a result of the Reviewing Editor and the peer reviewers coming together and discussing their recommendations until a consensus is reached. When revisions are invited, a fact-based synthesis statement explaining their decision and outlining what is needed to prepare a revision will be listed below. The following reviewer(s) agreed to reveal their identity: NONE.

no comment
